# Role of prior feeding status in mediating the effects of exercise on blood glucose kinetics

**DOI:** 10.1152/ajpcell.00271.2023

**Published:** 2023-08-29

**Authors:** Alfonso Moreno-Cabañas, Javier T. Gonzalez

**Affiliations:** ^1^Centre for Nutrition, Exercise and Metabolism, University of Bath, Bath, United Kingdom; ^2^Department for Health, University of Bath, Bath, United Kingdom; ^3^Exercise Physiology Lab at Toledo, University of Castilla-La Mancha, Toledo, Spain

**Keywords:** carbohydrate, flux, metabolism, physical activity, postprandial

## Abstract

Changes in blood glucose concentrations are underpinned by blood glucose kinetics (endogenous and exogenous glucose appearance rates and glucose disappearance rates). Exercise potently alters blood glucose kinetics and can thereby be used as a tool to control blood glucose concentration. However, most studies of exercise-induced changes in glucose kinetics are conducted in a fasted state, and therefore less is known about the effects of exercise on glucose kinetics when exercise is conducted in a postprandial state. Emerging evidence suggests that food intake prior to exercise can increase postprandial blood glucose flux compared with when meals are consumed after exercise, whereby both glucose appearance rates and disappearance rates are increased. The mechanisms underlying the mediating effect of exercise conducted in the fed versus the fasted state are yet to be fully elucidated. Current evidence demonstrates that exercise in the postprandial state increased glucose appearance rates due to both increased exogenous and endogenous appearance and may be due to changes in splanchnic blood flow, intestinal permeability, and/or hepatic glucose extraction. On the other hand, increased glucose disappearance rates after exercise in the fed state have been shown to be associated with increased intramuscular AMPK signaling via a mismatch between carbohydrate utilization and delivery. Due to differences in blood glucose kinetics and other physiological differences, studies conducted in the fasted state cannot be immediately translated to the fed state. Therefore, conducting studies in the fed state could improve the external validity of data pertaining to glucose kinetics and intramuscular signaling in response to nutrition and exercise.

## INTRODUCTION

The maintenance of blood glucose concentrations within a physiological range is of primary importance to human health. If blood glucose concentrations are too low (hypoglycemia), then individuals can become comatose; if too high for prolonged periods (hyperglycemia), then micro- and macrovascular damage can occur, increasing morbidity and mortality. Therefore, understanding the physiological mechanisms which underpin blood glucose concentrations can provide evidence to inform rational approaches to promoting and/or maintaining human health.

Blood glucose kinetics is the primary metabolic mechanism that controls blood glucose concentrations as rates of blood glucose appearance (glucose R_a_) and disappearance (glucose R_d_) govern blood glucose concentrations over time. Glucose R_a_ represents the summation of fluxes from endogenous glucose production (EGP) and exogenous glucose entering the circulation from the intestine, whereas R_d_ represents glucose exiting the circulation and entering tissues. Although many tissues contribute to R_d_—and the relative proportions will depend on the circumstances (discussed later)—the liver is the principal source of EGP in most physiological states (1). Thus, hepatic glucose production is crucial for systemic glucose homeostasis (2).

The preponderance of frequent meal eating and snacking in developed countries leads to a persistent postprandial state at meal times (3, 4) and during exercise (5). In contrast, glucose kinetics has predominantly been studied in a fasted state to maintain standardization, and this misrepresents the conditions in the free-living state and prevents direct generalization from the fasting state. The aim of this review is to compare glucose kinetics in response to exercise in fasting and postprandial states. It is noteworthy that essentially all of the evidence to date on glucose kinetics with exercise in the postprandial state is based on low-to-moderate intensity exercise and moderate-to-high carbohydrate diets. This may be (in part) due to difficulties in measuring glucose kinetics with multiple changes in fluxes [i.e., nonsteady state (6)], and also because moderate-to-high carbohydrate diets tend to be the most commonly consumed and are typically the recommendation from and health authorities. Accordingly, it should be noted that the responses described herein may not apply during conditions of high-intensity exercise or with low-carbohydrate diets, and future research could address those gaps.

## BLOOD GLUCOSE KINETICS DURING THE FASTED STATE

**Figure 1.**
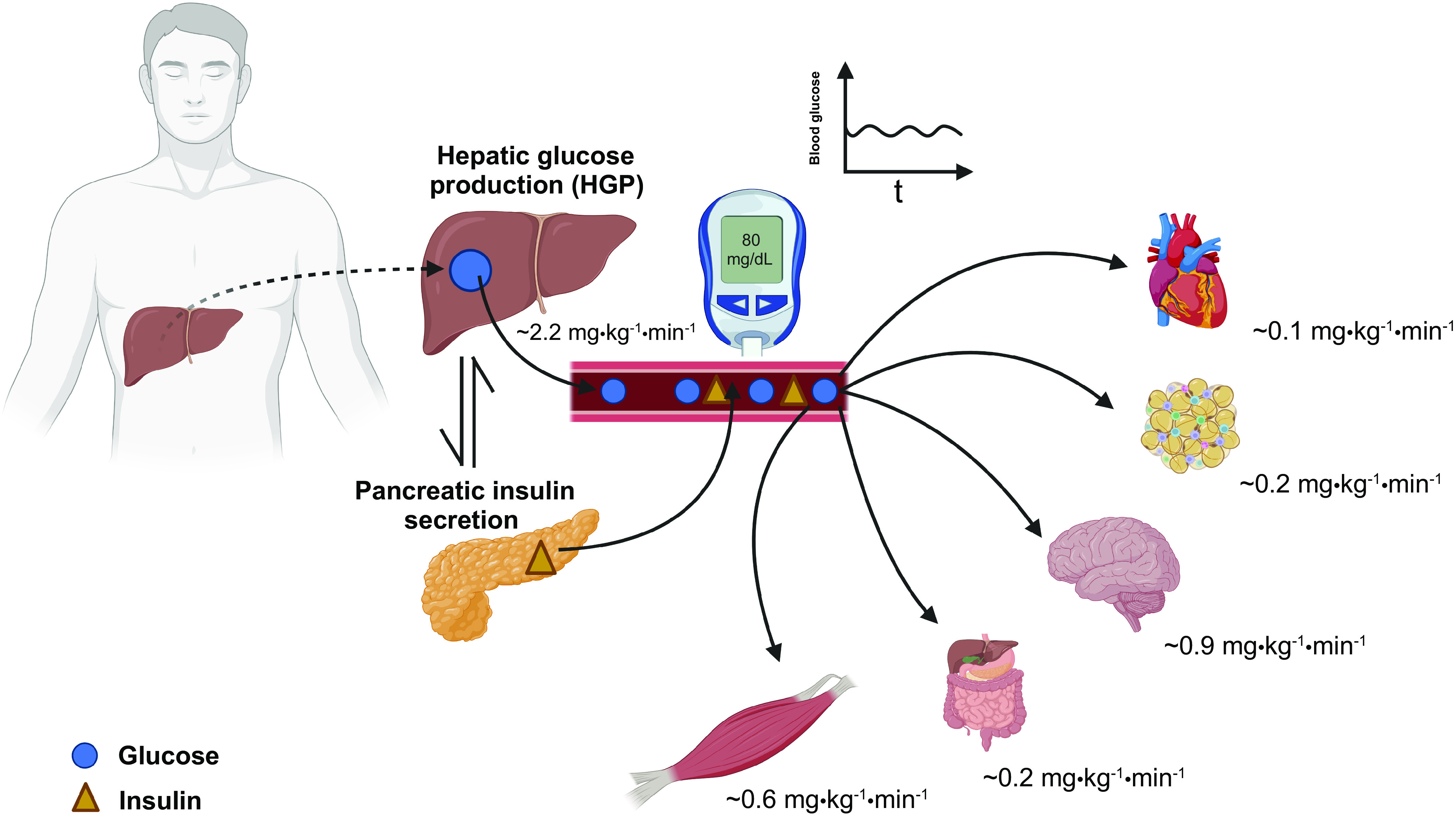
Glucose metabolism during the fasted state. Hepatic glucose production is the main source of glucose to maintain euglycemia and fuel glucose-consuming tissues (7), with insulin being a key regulator. Glucose (blue circle) and insulin (yellow triangle). Figure was created with BioRender.com and used with permission.

In the fasted state, blood glucose concentrations are relatively stable, and flux is ∼2.2 mg·kg^−1^·min^−1^. The liver provides glucose to maintain euglycemia and fuel obligate glucose-consuming cell types in various tissues [Fig. 1; (8)]. Glycogenolysis and gluconeogenesis each contribute ∼50% of total hepatic glucose production in humans up to the initial 24 h of fast (9). Glucose flux is decreased to ∼1.6 mg·kg^−1^·min^−1^ as the fast is extended to 46–64 h (9), mainly due to reduction in glycogenolysis. EGP is then primarily derived from gluconeogenesis although the absolute gluconeogenic rate is relatively constant over a range of physiological conditions (10). Insulin, glucagon, glucose, and glycogen itself are key regulators of hepatic glycogenolysis (2), with insulin being the principal activator of hepatic glycogen synthesis. Glucose is the primary suppressor of glycogenolysis, and glucagon activates hepatic glycogenolytic flux (2). Accordingly, with an initial fast, the low insulin-to-glucagon ratio and relatively low glucose concentrations result in net glycogenolysis. Then, as fasting becomes more prolonged and hepatic glycogen stores become depleted, the net rate of glycogenolysis decreases (9).

Under these conditions, glucose is mainly redistributed directly as a source of energy toward essential tissues that depend on the extracellular concentrations of glucose for survival [e.g., central nervous system and red blood cells (11)]. Skeletal muscle, on the other hand, derives its energy-yielding fuels from noncarbohydrate substrates, such as nonesterified fatty acids (NEFA) (12). The low insulin-to-glucagon ratio and the fasting hormonal response also lead to increased rates of lipolysis and the increased appearance of glycerol and NEFA from the adipose tissue. Therefore, this redistribution of the energy source as well as the increased plasma levels of NEFA decrease the glucose R_d_ in skeletal muscle.

## BLOOD GLUCOSE KINETICS DURING THE FED STATE

**Figure 2.**
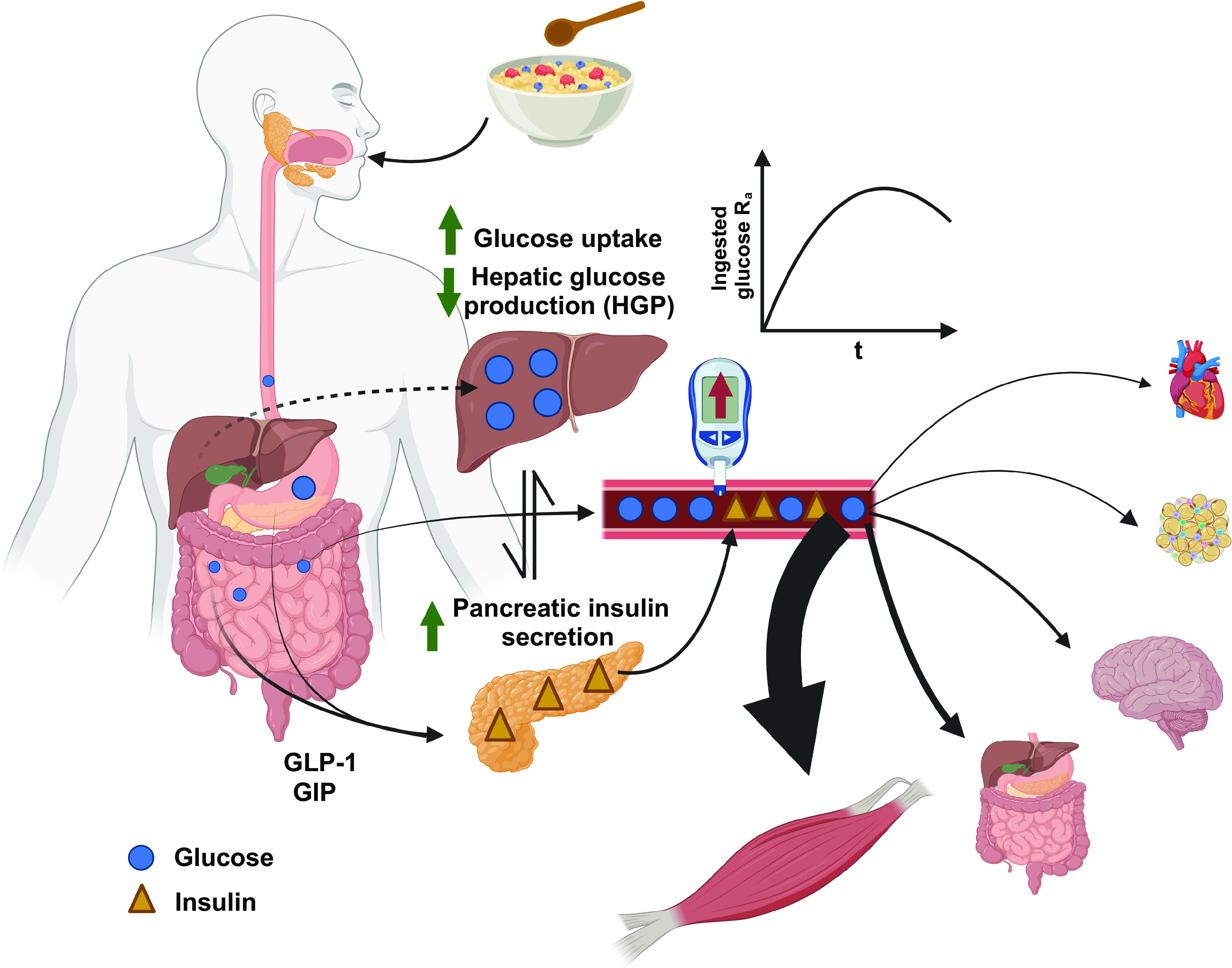
Postprandial glucose metabolism. Following digestion, blood glucose concentration increases by ingested glucose entering the circulation [i.e., exogenous glucose appearance (R_a_)] and increased secretion of insulin from the pancreas is stimulated. Due to the postprandial rise in the insulin and the gastrointestinal-derived hormones, the liver contributes to the rate of glucose disappearance (R_d_) of ingested glucose by increasing glucose uptake and suppressing hepatic glucose production. Insulin promotes glucose uptake into peripheral tissues such as muscle, which returns blood glucose to premeal concentration. Glucose (blue circle) and insulin (yellow triangle). GIP, glucose-dependent insulinotropic peptide; GLP-1, glucagon-like peptide-1. Figure was created with BioRender.com and used with permission.

In contrast to the fasted state where hepatic glucose production represents the main flux from glucose R_a_, in the fed state, ingested glucose typically becomes the primary source of glucose R_a_. This rapid increase in exogenous glucose R_a_ is a challenge to metabolic homeostasis and requires coordinated changes in the other components of glucose flux to buffer increases in blood glucose concentrations. The mechanisms by which blood glucose kinetics are regulated following carbohydrate ingestion include increases in gastric emptying, intestinal absorption, splanchnic and peripheral perfusion, and rates of tissue glucose uptake, which are all under some hormonal control (Fig. 2).

First, gastrointestinal-derived hormones play an important role after ingestion and the R_a_ of the glucose ingested would be influenced by the gastric emptying and intestinal absorption. In response to nutrient exposure, the enteroendocrine cells (i.e., K- and L-cells) secrete, two incretins, glucose-dependent insulinotropic peptide (GIP) and glucagon-like peptide-1 (GLP-1) (13, 14) that potentiate glucose-stimulated insulin secretion (15). Moreover, it is even suggested that physiological increases in GLP-1 augment glucose R_d_ and suppress EGP under hyperglycemic conditions (16). Therefore, direct contact of nutrients with intestinal cells induced by oral ingestion of food can influence insulin secretion and action, which increase glucose R_d_. The liver also contributes to the clearance from the blood of ingested glucose by increasing rates of glycogen synthesis and suppressing hepatic glucose production; these result in a switch from net hepatic glucose output to net hepatic glucose uptake (2).

The postprandial rise in insulin concentration facilitates glucose uptake into insulin-sensitive tissues, including skeletal muscle, adipose tissue, and the liver (17). Although it has been suggested that skeletal muscle is responsible for up to 90% of glucose disposal (18), this is largely estimated under unphysiological conditions, and the proportion of exogenous glucose disposed in skeletal muscle tissue is estimated to be ∼45%–50% with a mixed macronutrient diet (19). Insulin facilitates glucose uptake via several mechanisms, including tissue perfusion and translocation of the glucose transporter isoform 4 (GLUT4) to the cell membrane surface, allowing more glucose to enter the cell (20). In the absence of insulin, ∼90% of GLUT4 remains in intracellular vesicles (21). Transient increases in blood glucose concentrations are therefore restored to basal concentrations within 2–3 h following meal ingestion in most healthy people (22). Factors such as the quantity and glycemic index of the carbohydrate, as well as the nutrient composition of the meal, can influence the magnitude and duration of postprandial glycemia via effects on exogenous glucose R_a_, but also via effects on EGP and glucose R_d_ due to changes in insulinemia (23, 24). In addition, differences between and within people in their glycemic response to meals will also depend on hepatic and peripheral tissue insulin sensitivity, pancreatic β-cell insulin secretory capacity, and glucose effectiveness.

### The Second-Meal Phenomenon

Although blood glucose kinetics to single meals ingested after an overnight fast are relatively well characterized, the responses to sequential meals are less well documented and available evidence suggests several important differences with sequential meals versus single meals.

The second-meal phenomenon (25, 26) describes the improved glucose tolerance seen after the consumption of a prior glucose load. Glucose kinetics in two oral glucose tolerance tests (OGTTs) separated by 180 min, using a triple-tracer approach (27), has clarified the mechanisms underlying the second-meal effect. A greater suppression of hepatic glucose production, along with insulin potentiation, almost completely explains the second-meal effect observed, which was not influenced by exogenous glucose R_a_ and R_d_. There is evidence supporting the idea that the second-meal phenomenon is also explained by decreasing exogenous glucose R_a_ via slowing gastric emptying (28, 29) and enhanced glucose disposal via increased insulin sensitivity (30). Moreover, this is also supported by the evidence of greater muscle glycogen storage with the second-meal effect, in the presence of comparable insulinemia (31).

**Figure 3.**
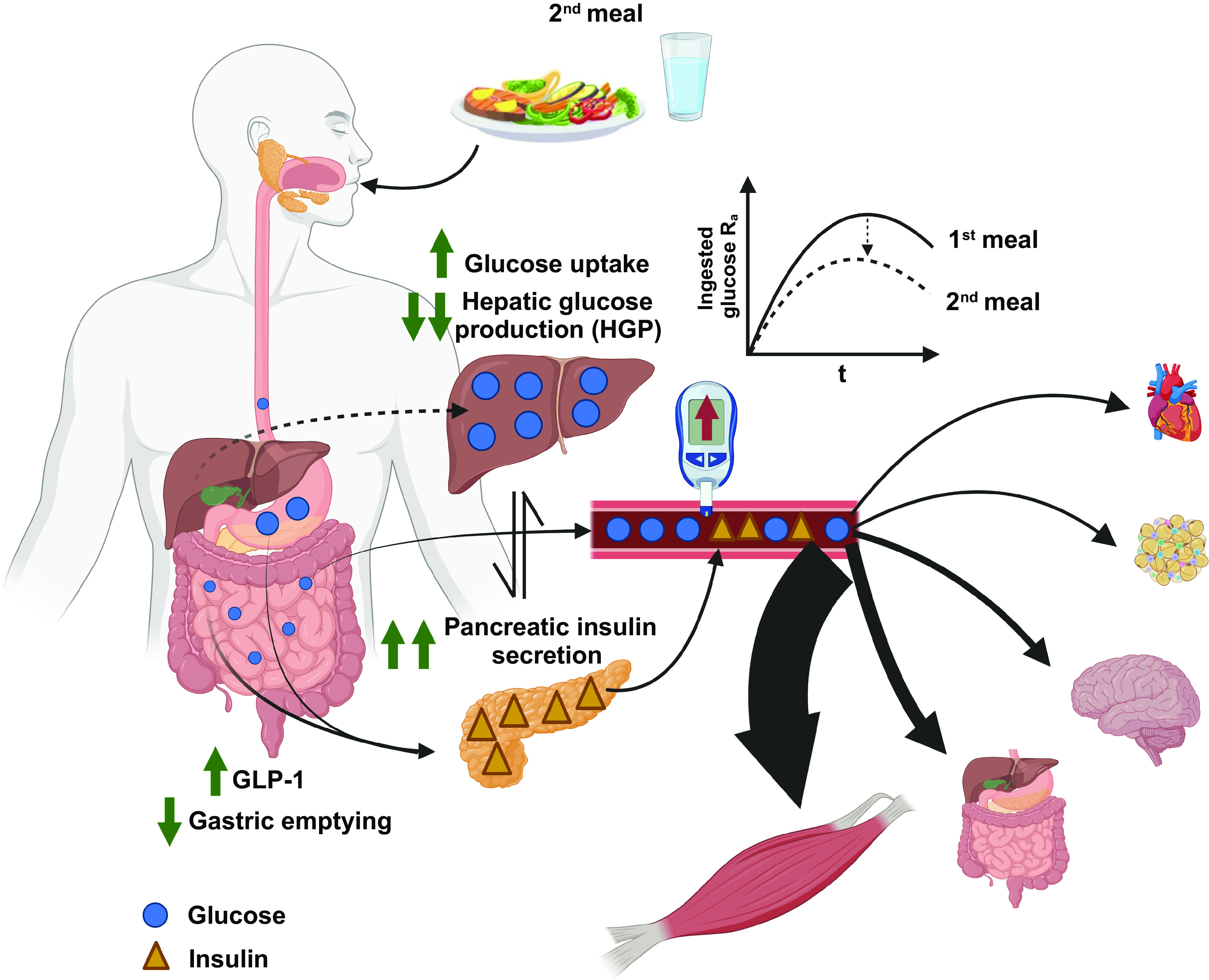
Second-meal phenomenon. Prior exposure to a meal delays gastric emptying of a subsequent meal with concomitant increases in gastrointestinal-derived hormones (e.g., GLP-1). This likely reduces ingested glucose entering the circulation (i.e., exogenous glucose appearance) and potentiates the early-phase insulin secretion. This potentiated insulin secretion combined with increased insulin sensitivity contributes to the further reduction in hepatic glucose production and enhanced muscle glucose uptake (R_d_). Glucose (blue circle) and insulin (yellow triangle). GLP-1, glucagon-like peptide-1. Figure was created with BioRender.com and used with permission.

Although this response is seen with mixed-macronutrient meals (28, 29, 31, 32), the second-meal effect is also dependent on the composition of the prior meal, and with more complex meal structures, other factors may come in to play. Prior consumption of fat or protein slows gastric emptying of a subsequent carbohydrate-rich meal, doubling the time to clear 50% of stomach content (28, 29). Both these studies also found greater postprandial responses of plasma GLP-1, which can slow gastric emptying (33) and potentiate insulin secretion (15). In addition, the potentiated insulin secretion induced by the enteroendocrine cells may depend on the prior and subsequent meal carbohydrate content. Potentiated early-phase insulinemia following sequential meals has been shown (34). This potentiation is likely due to priming of pancreatic β-cells by prior insulinemia exposure alongside suppressed pancreatic NEFA exposure and increased GLP-1 concentrations. Therefore, glucose tolerance is often improved with the ingestion of sequential meals, likely due to a slower gastric emptying (reducing exogenous glucose R_a_), combined with increased early-phase insulin secretion, reduced hepatic glucose production, and enhanced skeletal muscle glucose disposal (glucose R_d_; Fig. 3).

## BLOOD GLUCOSE KINETICS DURING EXERCISE

**Figure 4.**
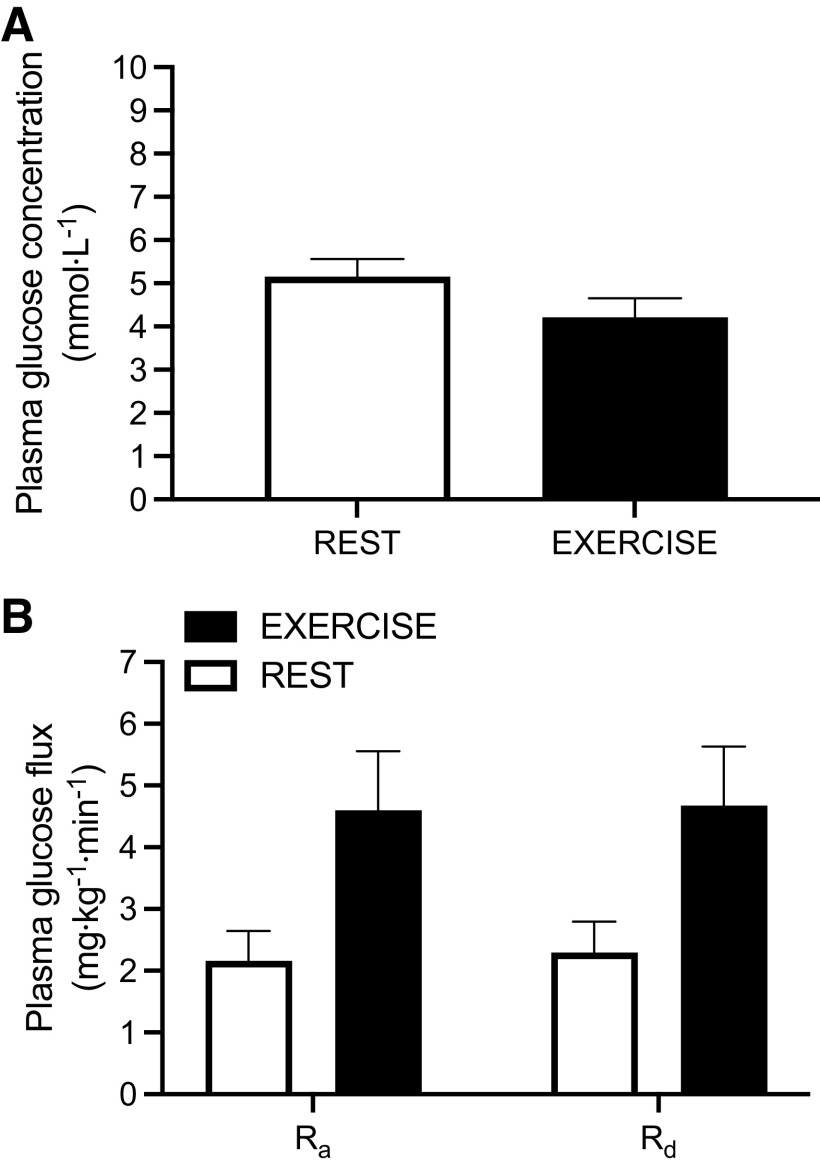
Plasma glucose concentration (*A*) and rate of appearance (R_a_) and disappearance (R_d_) of plasma glucose (*B*) during rest and moderate-intensity exercise (cycling at 50% W_max_) (35).

**Figure 5.**
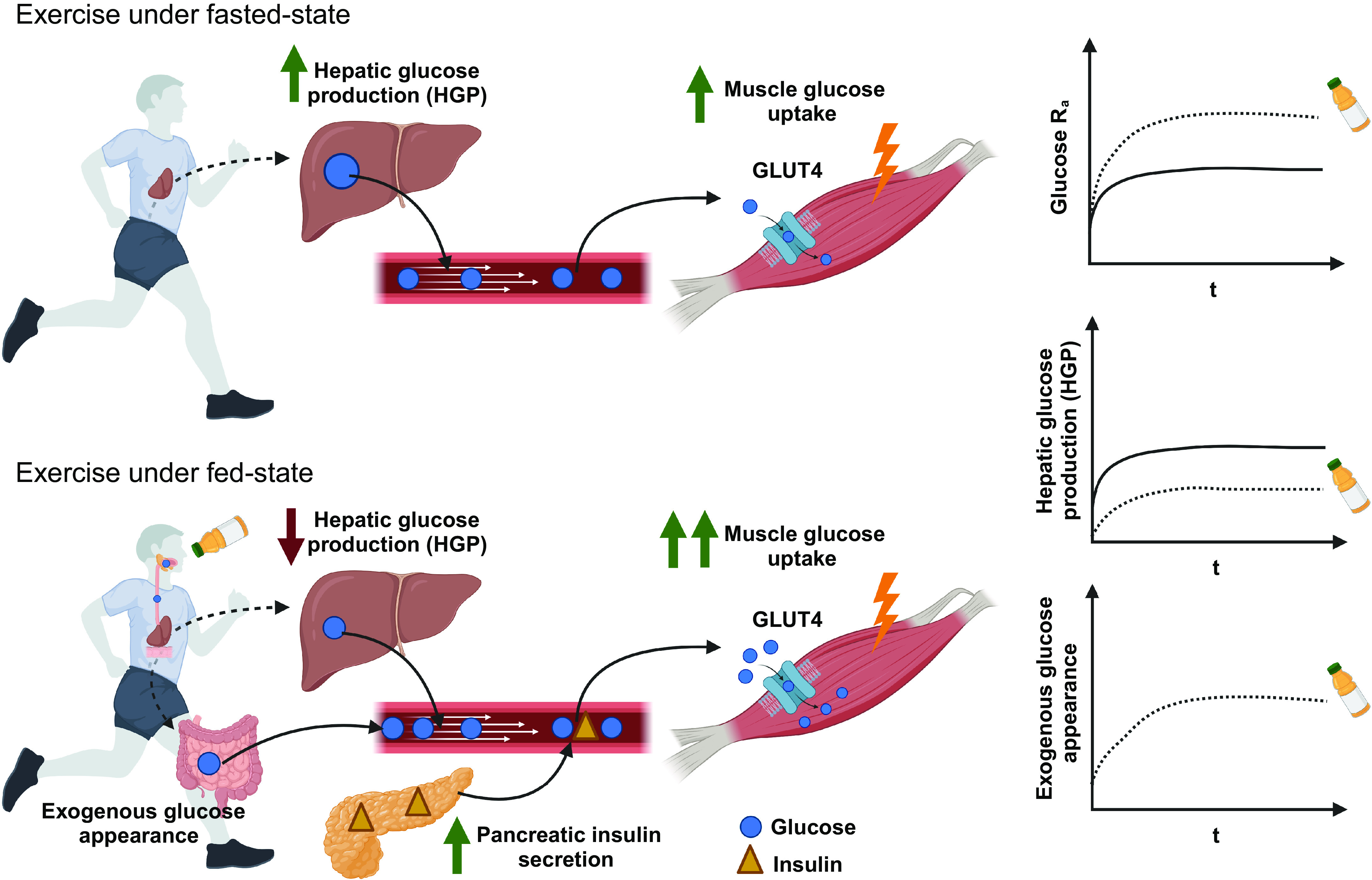
Glucose metabolism during exercise. In the fasted state, the increase in rate of appearance of plasma glucose (R_a_) is almost entirely due to an increase in hepatic glucose production. During exercise, GLUT4 translocation and activation facilitate muscle glucose uptake via insulin-independent mechanisms, supported by the increased blood flow to the active muscle. The rate of disappearance of plasma glucose (R_d_) would mainly represent glucose utilization by working muscle. In the fed state, the gut provides glucose to the bloodstream and exogenous glucose appearance contributes to the increased glucose R_a_. Glucose ingestion during exercise increases muscle glucose uptake (i.e., R_d_) and markedly decreases hepatic glucose production, supported by stimulation of both contraction and insulin-mediated glucose uptake. Glucose (blue circle) and insulin (yellow triangle). GLUT4, glucose transporter isoform 4. Figure was created with BioRender.com and used with permission.

Exercise drastically increases glucose flux, even without changing glucose concentrations. Moderate-intensity exercise at 50% W_max_ can increase glucose flux by >2-fold despite relatively little change in plasma glucose concentrations [Fig. 4; (35)]. Because glucose uptake by the brain (36), splanchnic bed (37), and resting muscle (38) remain relatively constant during exercise (or can even decrease), R_d_ would mainly represent glucose utilization by working muscle (39). To meet this increase in R_d_, and thus achieve maintenance of blood glucose concentrations, R_a_ is raised, primarily from increased hepatic glycogenolysis (Fig. 5).

The magnitude of increase in R_d_ due to exercise can be remarkable, as demonstrated by 20-fold increases in leg glucose uptake compared with rest (40). The underlying mechanisms driving increased R_d_ are multiple and include aspects of skeletal muscle glucose delivery, transport, and metabolism. During exercise, GLUT4 is translocated to the plasma membrane to facilitate glucose uptake via insulin-independent mechanisms (41, 42). Increased blood flow to the active muscle during exercise also supports the increase in muscle glucose uptake, and current hypotheses suggest that the intrinsic transporter activity of GLUT4 (43) or glucose-6-phosphate concentrations (44) may be increased to facilitate large increases in glucose uptake during exercise. Glucose-6-phoshate concentrations may increase during intense exercise (45) or with high muscle glycogen levels (44). The ensuing inhibition of hexokinase II may lead to accumulation of free glucose inside the cell and decrease the glucose gradient across the muscle cell (44), thereby decreasing rates of muscle glucose uptake. Since glucose uptake is the product of blood flow and the arteriovenous glucose difference, this increase in blood flow is quantitatively the larger contributor to the exercise-induced increase in muscle glucose uptake since the arteriovenous glucose difference only increases two- to fourfold during exercise (46). In addition to the large increase in bulk flow to contracting skeletal muscle during exercise, there is also the recruitment of capillaries that increases the available surface area for glucose delivery and exchange. Exercise-induced skeletal muscle glucose uptake then depends on the glucose delivery to the muscle capillaries, glucose transport out of the capillaries and into the cell, and glucose phosphorylation and subsequent further metabolism.

The increase in R_d_ during exercise is often offset by an increase in R_a_ that results in the maintenance of blood glucose concentrations. In the fasted state, the increase in R_a_ is almost entirely due to an increase in hepatic glycogenolysis (47). Hepatic glucose production is also increased in relation to exercise intensity (48, 49). Finally, glucose production by the liver decreases markedly when hepatic glycogen stores near depletion (50, 51), leading to an inability to maintain blood glucose homeostasis (often to hypoglycemic levels) (50–53), and an impaired performance/capacity in the absence of carbohydrate ingestion.

The gut provides glucose to the bloodstream when carbohydrate is ingested, and exogenous glucose appearance can be the main contributor to glucose R_a_ when glucose ingestion rates exceed ∼30 g/h (54). Glucose ingestion during exercise has minimal effects on net mixed muscle glycogen utilization (39, 53, 55), but increases muscle glucose uptake (i.e., R_d_) and markedly decreases hepatic glucose production (39, 56). Since both contraction and insulin-mediated glucose uptake are stimulated, there can be an additive effect on skeletal muscle glucose uptake and R_d_ (35, 57). The higher muscle glucose uptake and oxidation could be explained by the increases in arterial glucose availability (39, 56, 58), as well as a potential glucose-induced GLUT4 translocation (59). However, since metabolic clearance rate (i.e., glucose MCR = R_d_/glucose concentration) is also higher during exercise following carbohydrate ingestion, relatively higher plasma insulin (56) and lower plasma NEFA (60) could also contribute to the higher muscle glucose uptake.

## BLOOD GLUCOSE KINETICS FOLLOWING EXERCISE

Increased contraction-mediated muscle glucose uptake generally subsides within 3 h following exercise cessation. Subsequently, peripheral insulin sensitivity is enhanced via insulin-dependent mechanisms for up to 48 h following exercise (61). Improvements in insulin-stimulated muscle glucose uptake postexercise are attributed to glycogen resynthesis in glycogen-depleted muscle and also enhanced sensitivity of select proteins in the insulin signaling pathway (62). Both mechanisms are coordinated with increased microvascular perfusion for the enhanced insulin-stimulated muscle glucose uptake after exercise (63). Muscle glycogen synthase activity is increased postexercise to promote glycogen resynthesis (64), raising muscle glucose disposal in proportion to the glycogen used during exercise (65). Furthermore, a distal protein in the insulin signaling cascade, Akt substrate of 160 kDa (AS160), is activated by exercise and it is associated with increased insulin-stimulated glucose uptake in the postexercise period (66). Finally, there is emerging evidence to suggest that acute exercise redistributes intracellular GLUT4 that facilitates the recruitment in muscle (67) and enhances muscle membrane permeability to glucose in the postexercise period (68).

Due to this elevated postexercise increase in R_d_ glucose (i.e., muscle glucose uptake), oral glucose tolerance should be logically improved following exercise. Nevertheless, there is evidence that one bout of endurance exercise tends to reduce (69–72) or not affect significantly (73, 74) oral glucose tolerance in healthy individuals. It has been demonstrated that postprandial blood glucose concentrations are not lowered immediately after exercise, because postexercise, the increase in postprandial glucose R_d_ can be offset by increases in both endogenous and meal-derived glucose R_a_ (72, 75). Since changes in gastric-emptying rates are not typically evident postexercise (76), the greater rates of exogenous glucose R_a_ could represent changes in perfusion and/or intestinal permeability. Small intestine permeability can be increased following exercise (70% W_max_), possibly due to intestinal cell damage (77). Once glucose is orally ingested following exercise [70% of maximal oxygen uptake; (78)], intestinal perfusion can be increased by 15%–35%, which is associated with intestinal glucose absorption (79). Combined with this effect on intestinal perfusion, other factors such as catecholamines (80) and gastrointestinal hormone responses [e.g., GLP-1 and GIP; (70)] could contribute to changes in exogenous glucose R_a_. Importantly, this response appears to be physiological rather than pathophysiological, as greater increase in R_a_ than R_d_ seems to only be observed in people with normal glucose tolerance, whereas in people with type 2 diabetes, the exercise-induced increase in R_d_ is greater than the increase in R_a_ (75).

### Feeding Prior to Exercise

Although studies where a bout of exercise has been conducted in the fasted state suggest that increases in glucose R_d_ postexercise can be offset by increases in glucose R_a_ postexercise, this may not translate to other nutritional states. As mentioned, people consume food and perform exercise while still in a postprandial period from a prior meal during their daily living (4, 5). Therefore, it is relevant to assess whether a prior meal (preexercise) affects the exercise-induced changes in glucose kinetics after exercise.

When a 75-g oral glucose tolerance test was administered immediately after 60 min of moderate-intensity exercise, consumption of a prior high-carbohydrate meal (57% of energy intake as carbohydrate; high glycemic index) before exercise led to a further increase in both glucose R_a_ and R_d_ (35). Plasma glucose flux during the OGTT was thus ∼25%–50% higher with prior breakfast consumption before exercise with relatively little change in glucose concentrations. The increase in glucose R_a_ was almost entirely explained by increased exogenous glucose R_a_. The mechanisms contributing to the increase in glucose flux are currently unknown, but it is possible that changes in splanchnic blood flow, intestinal integrity, splanchnic glucose extraction, and/or intramuscular signaling all play a role. For example, consumption of a meal (60% of energy as carbohydrate and 20% of energy as lipid and protein each) prior to exercise (55% of maximal oxygen uptake) results in greater splanchnic blood flow during exercise, maintaining greater availability of exogenous glucose to appear in the circulation (76). The role of intestinal integrity is less clear, since markers of intestinal damage decrease with high-carbohydrate meal consumption before exercise (35). In addition, there is some evidence that macronutrient composition interacts with exercise to alter the incretin hormone response (e.g., GIP) with sequential meals (81). Future studies should, however, control for time of day to better understand the effects of meal pattern per se in the context of lower versus higher carbohydrate diets. Therefore, the mechanisms underlying changes in glucose R_a_ in response to feeding before exercise require further examination.

**Figure 6.**
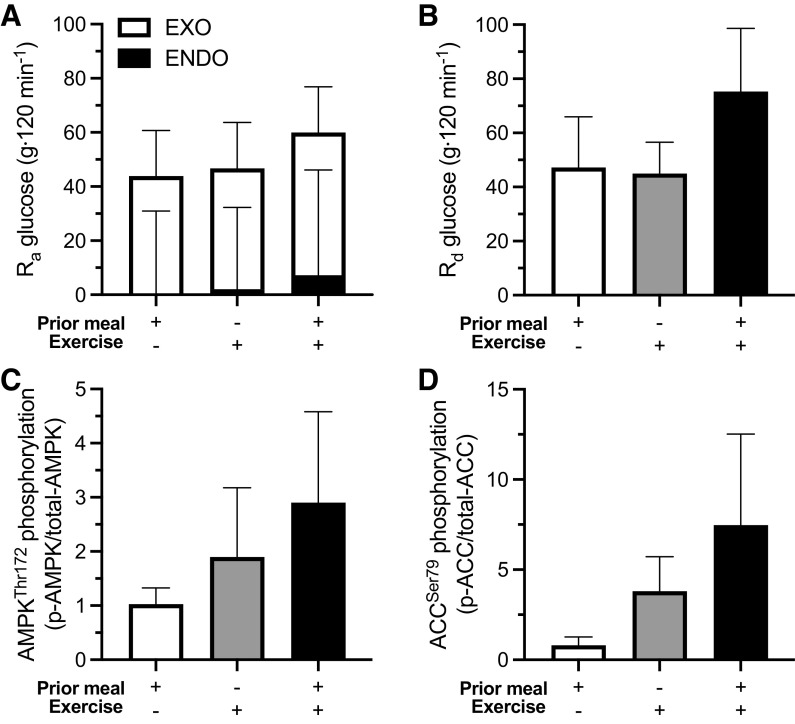
Postprandial rate of appearance (R_a_; exogenous and endogenous glucose appearance; *A*) and disappearance (R_d_; *B*) of plasma glucose, during an oral glucose tolerance test (OGTT) after overnight fasted state or following exercise in the fed/fasted state. Phosphorylation of AMPK^Thr172^ (*C*) and ACC^Ser79^ (*D*) after overnight fasted state or following exercise in the fed/fasted state (35).

In contrast to the mechanisms underlying glucose R_a_ following preexercise feeding, more evidence is available on the potential mechanisms contributing to glucose R_d_. In the same study demonstrating increased postexercise glucose R_d_ with preexercise feeding, intramuscular activation of Adenosine 5′-monophosphate-activated protein kinase (AMPK) was also increased, as indicated by increased phosphorylation of AMPK^Thr172^ and Acetyl-CoA carboxylase (ACC)^Ser79^ (Fig. 6). AMPK can potentiate postexercise skeletal muscle glucose uptake via stimulating GLUT4 translocation. Although the increase in AMPK activity is therefore consistent with the hypothesis that increased AMPK activity contributed to the increase R_d_ glucose, it may be surprising that a carbohydrate-rich meal consumed before exercise produces such an effect on AMPK activation. AMPK activation is regulated not only by the energy status of the cell but also by the glycogen concentration. AMPK contains a glycogen binding domain on the beta subunit, and glycogen can allosterically inhibit AMPK activity (82).

It might be expected that a high-carbohydrate, preexercise meal would result in a higher muscle glycogen concentration and therefore decrease, not increase, postexercise AMPK activity. Following ingestion of a carbohydrate-rich meal, however, muscle glycogen concentrations can first decrease, before starting to show a net increase by 2–3 h later postmeal (83). The most likely explanation for this is due to the time course of changes in glucose flux. The increase in insulin secretion rates are rapid and detectable within 10 min of an OGTT. This increase in insulin can stimulate carbohydrate utilization within muscle, whereas the time course for exogenous glucose to appear in the circulation and be taken up by muscle can be slower (27). Accordingly, in the early postprandial period, there can be a mismatch between muscle glucose utilization and delivery, which would explain a net decrease in muscle glycogen concentration. It is therefore possible that commencing exercise at this timepoint after a meal can result in a high AMPK activity compared with exercise in the fasted state via a mismatch between carbohydrate utilization and delivery. Consistent with this hypothesis, in the study demonstrating increased skeletal muscle AMPK activity with preexercise feeding versus preexercise fasting, the increase in whole-body carbohydrate utilization (124 ± 138 g) was almost double the carbohydrate provided by the preexercise meal (65 g).

## FUTURE DIRECTIONS

There are several key avenues that future research could take to enhance knowledge in this area. Current evidence suggests that carbohydrate intake before and/or during exercise within a training program could blunt the increases in oral glucose insulin sensitivity (84, 85). Nevertheless, it is unclear whether this blunting effect was explained by an adaptation of increased glucose R_a_ (exogenous or/and endogenous) or decreased glucose R_d_. In addition, further research is needed to clarify adaptations in glucose flux during exercise after a fed or fasted training program. Postprandial insulin sensitivity could be even deteriorated in an intensity-dependent manner immediately after exercise due to hormonal changes [i.e., catecholamines (80)]. Higher intensities may alter glucose R_a_/R_d_ during exercise in the postprandial state, and further research is therefore needed on the effects of exercise intensity on blood glucose kinetics. Furthermore, since there may be sex and/or body size differences in glucose kinetics (86), the role of sex and body size in response to sequential meals with and without exercise could be better understood. Finally, the baseline insulin sensitivity status may alter the response to postexercise glucose tolerance (73), yet the role of baseline insulin sensitivity status on effects of preexercise food intake and on postexercise metabolism would benefit from further research.

## CONCLUSIONS

Improved glucose tolerance seen after the consumption of a prior meal is known as the second-meal phenomenon and is characterized by decreased endogenous and exogenous glucose appearance rates, combined with enhanced insulin sensitivity and glucose disappearance rates. Exercise (moderate intensity) conducted in a fasted state can also increase the postprandial insulin sensitivity and glucose disappearance rates, but this can be offset by increases in endogenous and exogenous glucose appearance rates. When exercise is conducted in a fed state, postprandial glucose kinetics after exercise can show a further increase in flux compared with exercise in a fasted state. Increased glucose appearance rates could be due to changes in splanchnic blood flow, intestinal integrity, and/or splanchnic glucose extraction, meanwhile, increased glucose disappearance rates could be explained by increased activation of AMPK, which can stimulate GLUT4 translocation and thus potentiate postexercise skeletal muscle glucose uptake. Therefore, current evidence suggests that food intake before exercise alters blood glucose kinetics after exercise, and evidence from exercise in the fasted state cannot be immediately translated to exercise in the fed state.

## GRANTS

This work was funded by British Heart Foundation PG/19/43/34432 (to J.T.G.).

## DISCLOSURES

J.T.G. has received research funding from Biotechnology and Biological Sciences Research Council (BBSRC), Medical Research Council (MRC), British Heart Foundation, Clasado Biosciences, Lucozade Ribena Suntory, ARLA Foods Ingredients and Cosun Nutrition Center. J.T.G. is a scientific advisory board member to ZOE and 6d Sports Nutrition and has completed paid consultancy for The Dairy Council, PepsiCo, Violicom Medical, Tour Racing Ltd., and SVGC.

## AUTHOR CONTRIBUTIONS

A.M.-C. and J.T.G. conceived and designed research; A.M.-C. and J.T.G. performed experiments; A.M.-C. and J.T.G. analyzed data; A.M.-C. and J.T.G. interpreted results of experiments; A.M.-C. and J.T.G. prepared figures; A.M.-C. and J.T.G. drafted manuscript; A.M.-C. and J.T.G. edited and revised manuscript; A.M.-C. and J.T.G. approved final version of manuscript.
